# Influenza vaccine: a review on current scenario and future prospects

**DOI:** 10.1186/s43141-023-00581-y

**Published:** 2023-11-30

**Authors:** Dipanshi Gupta, Sumedha Mohan

**Affiliations:** Amity Institute of Biotechnology, Amity University Uttar Pradesh (AUUP), Sector-125, Noida, Uttar Pradesh 201303 India

**Keywords:** Influenza vaccine, Recombinant technology, Immune response

## Abstract

Vaccination is a crucial tool in preventing influenza, but it requires annual updates in vaccine composition due to the ever-changing nature of the flu virus. While healthcare and economic burdens have reduced, the virus remains a challenge. Research conducted over the past decade has revealed pathways for improvement through both basic and clinical studies. Viral surveillance plays a vital role in the better selection of candidate viruses for vaccines and the early detection of drug-resistant strains.

This page offers a description of future vaccine developments and an overview of current vaccine options. In the coming years, we anticipate significant changes in vaccine production, moving away from traditional egg-based methods towards innovative technologies such as DNA and RNA vaccines. These newer approaches offer significant advantages over traditional egg-based and cell culture-based influenza vaccine manufacturing.

## Description

“Influenza virus is an acute respiratory disease that significantly impacts human well-being. Commonly referred to as seasonal flu, it poses a continuous challenge for vaccine development. There are three main methods for creating influenza vaccines: inactivated influenza vaccine, live attenuated influenza vaccine, and recombinant hemagglutinin (HA) influenza vaccine. Influenza has a high mutation rate, which necessitates the production of new recombinant vaccines twice a year to mitigate its spread. Global health organizations like the World Health Organization (WHO) and other stakeholders convene biannually to assess the virus’s prevalence and recommend suitable vaccines for worldwide use.

Vaccine development typically involves two categories: trivalent and quadrivalent vaccines. Trivalent vaccines include two strains of influenza A and one strain of influenza B. They are primarily produced in chicken eggs. In contrast, quadrivalent vaccines contain two strains of influenza A and two strains of influenza B and are developed using recombinant techniques in the laboratory. Efforts are underway to create a more effective and comprehensive vaccine known as the universal influenza vaccine.”

## Background

The viruses responsible for pandemic influenza are believed to originate from wild waterfowl, serving as the primary natural source of influenza viruses. Reassortment is the process through which genetic material from human-infectious virus strains is periodically exchanged with genetic material from avian virus strains.

The first inactivated flu vaccine was developed by Thomas Francis and Jonas Salk at the University of Michigan, with assistance from the US Army. Prior to its approval for widespread use in 1945, the vaccine underwent safety and effectiveness testing on members of the US military.

The aims of this article are to prevent the need for seasonal vaccine reformulation and to explore the various types of vaccines available worldwide.

## Introduction

The seasonal influenza virus is a severe respiratory viral illness caused by influenza viruses that are distributed worldwide. Influenza viruses are classified into four types: type A, type B, type C, and type D. The most life-threatening type is Influenza A, which can cause death and various respiratory diseases. Influenza type B viruses are also responsible for seasonal flu pandemics in humans. The seasonal flu vaccines, such as B/Victoria and influenza B/Yamagata, target the two lineages that are currently circulating [1]. Influenza C viruses are not known to cause epidemics and typically induce milder flu symptoms. Influenza D viruses have been found to infect animals such as pigs, cattle, and sheep but not humans [2].

Several pandemics and annual epidemics have occurred periodically in recent decades, with significant negative impacts on the global economy and public health. Influenza viruses belong to the Orthomyxoviridae family, which includes enveloped RNA viruses. These viruses have an eight-segmented single-stranded negative-sense RNA genome, which can be divided into either seven (subtypes C and D) or eight (subtypes A and B) distinct segments [3]. These segments encode various proteins, including Hemagglutinin (HA), Neuraminidase (NA), nucleoprotein (NP), matrix protein 1 (M1) and (M2), polymerase subunits (PA, PB1, and PB2), non-structural proteins, nuclear export proteins, and more recently identified proteins such as PBI, M42, PB1-F2, and PA-X [4].

Influenza causes significant illness and fatalities worldwide each year. Vaccines are essential for both preventing future epidemics and managing current ones. Vaccines have been in use for over 60 years, as they help bolster an organism’s immune system. Over time, immunity to the virus naturally decreases, which is why an annual influenza vaccination is recommended. The effectiveness of seasonal vaccinations depends on the degree of match between the vaccine strains and the prevalent strains in the population [5]. The ability of the virus to change its genetic makeup allows it to evade immunity and infect hosts again. Major flu pandemics are typically triggered by antigenic shifts, significant changes in the virus’ antigenic properties resulting from genetic rearrangements involving two co-infecting subtype strains [6].

Several challenges can make vaccination programs less effective, including the need to predict which strains will be common each season, the unpredictability of the virus, and the potential lack of innate immunity to new strains within the population. Influenza vaccines are most effective when the vaccine virus closely matches the circulating viral strain [7]. To address the continuous evolution of influenza viruses, the World Health Organization (WHO) and its committee members hold biannual meetings to determine the components of the influenza vaccine.

Traditional vaccines have limitations, emphasizing the importance of diverse vaccine platforms. Molecular engineering tools expedite innovative vaccine development, ultimately enhancing disease protection [8]. Gene and protein engineering, in addition to viral-vectored vaccines, present promising approaches to enhance immunization strategies. Exploration of more efficient delivery methods has the potential to lead to further breakthroughs in vaccine development. The least invasive, cost-effective strategy for guarding against influenza virus infections is vaccination. Currently, three types of seasonal influenza vaccines are available: inactivated influenza vaccines produced from eggs and cells, live attenuated influenza vaccines, and baculovirus recombinant HA vaccines manufactured in insect cells (as listed in Table 1). Nevertheless, various limitations persist in terms of both vaccine availability and effectiveness. Therefore, innovative technologies in the field of influenza vaccine formulation and production may hold the key to addressing the limitations of current influenza vaccines. This article presents an overview of the current vaccination status and promising research in the development of next-generation vaccines.

## Objective

The primary goal is to raise awareness of the nature of the influenza virus and the number of vaccines available to fight against it, as this is a common issue. Numerous vaccines, including recombinant HA vaccine, inactivated vaccine, and live attenuated vaccine, are already being developed worldwide and are on the market. The WHO and other relevant parties certify each of these vaccinations. Other vaccines, referred to as next-generation vaccines, aim to improve durability, strengthen resistance to variation, and prevent influenza virus infection by utilizing particle vaccine technology. Current developments to boost vaccination efficacy include universal influenza vaccines and vaccines based on nanoparticles; all of these vaccines require yearly updates.

## Current vaccines against influenza

Globally, seasonal influenza epidemics are expected to cause 290,000 to 650,000 respiratory fatalities each year and 3 to 5 million cases of severe illness [9]. The influenza virus, commonly known as the seasonal flu, can be influenced by environmental and weather factors. The virus can evade the human immune system due to unique mutations introduced within the surface glycoproteins HA and NA. Neither spontaneous infection nor vaccination provides lifelong protection against the virus. Antigenic drift, the small changes in antigenicity of the influenza virus, is a frequent cause of influenza epidemics [10–12].

Currently, three types of vaccine development platforms are globally employed: inactivated influenza vaccine, live-attenuated influenza vaccine, and recombinant-HA vaccine. These vaccines have their advantages and disadvantages. Vaccines can activate cellular and immunological effectors, including antibodies produced by B lymphocytes that can bind specifically to viral antigens and cytotoxic CD8+ T-lymphocytes, which destroy infectious cells and prevent their spread in the body. Since protection from the flu is only temporary [13], all vaccines must be regularly updated to match the evolving strains of the virus that are currently circulating [14, 15].

In order to select the most appropriate influenza vaccine, biannual meetings are held during which the WHO and other stakeholders recommend suitable influenza vaccines for global use. These recommendations are based on genetic and antigenic features, as well as epidemiological data from different countries, which are compared to viruses circulating worldwide [16, 17].

Various manufacturers currently offer trivalent vaccines and the most recent quadrivalent vaccines on the market. For many years, trivalent vaccines have been developed to provide protection against three different influenza viruses. These formulations include one lineage of influenza B viruses and two lineages of influenza A (H1N1 and H3N2) viruses. Additionally, there are two different lineages of B virus circulating, so the second lineage of B virus is used in the formulation of the quadrivalent influenza vaccine, which can provide protection against the current virus mutations. Currently, officially approved influenza vaccines are significantly standardized in terms of HA content and immunogenicity (Table 1).

**Table 1:** Some of the influenza vaccines which are licensed and currently present worldwide

Vaccine name	Manufacturer	Country
**Influenza vaccine [in-activated vaccines], split virion, egg-based vaccines**		
**(AQ) Afluria-quadrivalent**	Seqirus-Pty. Ltd.	USA
**(FQ) Fluarix-quadrivalent**	Glaxo-SmithKline Biologicals	USA
**(FLQ) FluLaval-quadrivalent**	ID-Biomedical Corporation of Quebec Sanofi Pasteur, Inc.	USA
**(FHQ) Fluzone-highdose quadrivalent**	Sanofi Pasteur, Inc.	USA
**(FQ) Fluzone quadrivalent**	Sanofi Pasteur, Inc.	USA
**(FQ) FluQuadri**	Sanofi-Aventis Australia	Australia
**(VT) Vaxigrip tetra**	Sanofi-Aventis Australia	Australia
**(FT) Fluarix tetra**	Glaxo-SmithKline Biologicals	Australia
**(AQ) Afluria quadrivalent**	Seqirus Pty. Ltd.	Australia
**(IFT) Influvac tetra**	Mylan Health	Australia
**Influgen**	Lupin Laboratories Ltd.	India
**Influenza vaccine [in-activated vaccines], surface antigen, adjuvanted, egg-based**		
**(FQ) Fluad-quadrivalent**	Seqirus, Inc.	USA
**Agripal**	Chiron Panacea Vaccines Pvt. Ltd.	India
**(RIV) Recombinant influenza vaccines**		
**Flublok-quadrivalent**	Sanofi-Pasteur, Inc.	USA
**(CSV) Cadiflu-S vaccine**	CPL Biologicals Pvt Ltd.	India
**(SIV) Subunit influenza vaccine [in-activated vaccines], cell culture-based vaccines**		
**Flucelvax-quadrivalent**	Seqirus, Inc.	USA
**(LAIV) Live-attenuated influenza vaccine**		
**(FMQ) Flu-mist-quadrivalent**	Med-Immune, LLC.	USA
**(NSV) Nasovac S vaccine**	Serum Institute of India Ltd.	India

## References: [18–22]

### In-activated influenza vaccine

Inactivated virus-based vaccination is the most commonly used technique due to its low production costs and high safety levels. In this method of vaccination, the virus is typically produced in cultured mammalian cells and embryonated chicken eggs. While booster shots may be required to maintain antibody titers, it has been previously demonstrated that inactivated influenza vaccines can generate systemic immunity [23]. There are three different types of inactivated influenza vaccines: Split-Virus Inactivated Vaccines, Whole-Virus Inactivated Vaccines, and Subunit Inactivated Vaccines.

In split-virion vaccination, the viral envelope of the whole virion is disrupted through diethyl ether or detergent treatment. Whole-virion vaccines can be produced by chemically inactivating viruses using formaldehyde or -propiolactone. Subunit vaccines are composed of further refined HA and NA by removing all the envelope. Since the initial isolates of the A (H3N2) virus do not replicate well in eggs, inactivated vaccines require adaptation in eggs to achieve significant cytotoxicity. Excessive passages in eggs may lead to a mismatch in antigenic drift between HA and the epidemic isolates [24–26]. To prevent egg-adaptive changes in HA, viral adaptation and replication can be achieved using cultured-cell lines, such as Madin-Darby canine kidney and Vero Cells [27]. However, the vaccine seed virus titers in these suspension-grown cell lines in fermenters often lower egg titers, resulting in increased costs and reduced output [28]."

### Live-attenuated influenza vaccine’s

Both Immunoglobulin A (IgA) and Immunoglobulin G (IgG play significant roles in the immune system. IgA is the primary isotype found in mucous membrane secretions, primarily functioning on epithelial cell surfaces. IgG, on the other hand, is the primary isotype in extracellular fluids and blood, primarily functioning within the tissues of the human body. Live attenuated vaccines can stimulate the production of both IgA and IgG in the upper respiratory tract, where the virus initially replicates. This immune response can lead to cross-reactions [29, 30]. However, individuals with specific underlying disorders or compromised immune systems are not advised to receive live attenuated vaccines. In such cases, the virus can replicate itself and cause larger infections within the individual, potentially resulting in adverse outcomes.

### Recombinant–hemagglutinin (HA) vaccine

Recombinant HA vaccines demonstrate economic viability and yield impressive results. Several manufacturers have already adopted recombinant protein expression platforms based on insect cells and baculovirus for producing these vaccines [31]. Unlike egg-based influenza vaccines, recombinant HA vaccines do not exhibit unwanted mutations, offering a significant advantage.

Additionally, HA recombinant vaccines can be formulated in less than two months, making them well-suited for preventing pandemic influenza viruses [32]. Although inactivated vaccines and HA vaccines share the same mechanism of action, commercially available recombinant HA vaccines contain three times more HA than inactivated influenza vaccines while generating antibodies comparable to traditional inactivated vaccines [33]. However, it is important to note that HA recombinant vaccines are limited to the age group of individuals aged 18 and above [34]. Sanofi Pasteur’s Flublok Quadrivalent is the first HA recombinant vaccine available.

## Vaccine platforms: represent the next generation vaccines

### Virus-like-particle vaccines

The term “virus-like particle” refers to non-replicating particles that mimic viruses and are composed of surface viral glycoproteins. These VLPs lack virus genetic material, yet they imitate the original viral shape of the virus and cannot induce infection. VLPs have been reported as effective vaccine candidates and have been used to develop effective vaccines against many viruses, such as the Hepatitis-B virus, including vaccines like Heptavax-B, Engerix-B (produced by GlaxoSmithKline Biologicals, Rixensart, Belgium), and Hepavax-Gene (produced by Janssen Vaccines Corp., Incheon, South Korea). They have also been used for human papillomavirus vaccines like Gardasil, Cecolin, Gardasil-9, and Cervarix (all produced by GlaxoSmithKline Biologicals), as well as for vaccines against the hepatitis E virus (HEV) [35]. As a result, it has been demonstrated that the VLP platform is a promising technique for producing vaccines, particularly for influenza. Innate immunity can be activated by dendritic cells and antigen-presenting macrophages, leading to the well-organized generation of virus-specific T cell and B cell responses. Influenza vaccines built on the VLP platform may combine several viral proteins like neuraminidase (NA), hemagglutinin (HA), M2e, or M1 into a single VLP, providing greater flexibility in vaccine formulation and a broader spectrum of protection [36].

A comprehensive and well-balanced immune response can be induced by adding viral neuraminidase to the common influenza vaccine [37]. M2e, on the other hand, may be the focus of future universal vaccinations. The VLPs composed of M1 and NA triggered immune responses with significant immunity and cross-reactivity against heterologous and homologous antigens, particularly for influenza A subtypes [38]. There are currently pre-clinical or clinical trials being conducted on certain influenza vaccine candidates built on the VLP platform [39–41]. Phase 2 clinical studies evaluating the safety and immunogenicity of the recombinant VLP vaccine from Novavax were conducted in 2012 [42]. This influenza VLP vaccine produced a robust immune response after just one injection, generating strong antigen-specific CD4+ T cells and antibody responses in response to the plant-based VLP influenza vaccination. Phase 2 clinical studies for plant-based VLP (HA) vaccines were completed by Medicago in 2019 [43]. These VLP vaccination candidates generated both cellular and humoral immune responses, resulting in the production of antibodies against four distinct influenza strains (two influenza A and two influenza B), along with cross-protection.

### Nanoparticle-based influenza vaccines

Conventional influenza vaccines provide complete protection against the influenza virus. However, protection against influenza in the mucosal respiratory system [44, 45] is also advantageous. Nanoparticles are suitable for enhancing mucosal immunity due to their solubility and stability, which help defend against respiratory infections. These nanoparticles function as carriers for antigens and can be made from organic or synthetic materials [46].

Previously, papaya mosaic virus nanoparticles were used as an adjuvant in combination with trivalent inactivated influenza vaccines to investigate their effectiveness in mice. The study, based on antibody titers such as IgG, IgA, and IgG2 in bronchoalveolar lavage samples and blood, demonstrated that this nanoparticle vaccine was superior in inducing anti-influenza immunity, especially after intranasal immunization [47]. Furthermore, a polylactic-co-glycolic acid nanoparticle combined with influenza A (H1N1) conserved peptides was used for intranasal immunization, which was shown to protect the lungs of pigs. It was demonstrated that this immunization could activate CD4+ and CD8+ T lymphocytes specific to the antigen [48].

To improve defense against the influenza virus and produce cross-protective antibodies that target multiple infection-related pathways, Helix C and the ectodomain of the matrix protein 2 were combined to create a self-assembled nanoparticle. This nanoparticle was then utilized to combine two separate conserved influenza antigens, successfully eliciting neutralizing antibodies in mouse models [49].

## Limitations

The fact that certain nanoparticles in vaccines are cytotoxic poses several limitations. Additionally, there are drawbacks to the nanoparticle-based vaccine delivery system. For instance, when discussing virus-like particles, the size of the polydisperse particles can vary, and there is a lack of repeatability in producing VLPs. In a vaccine system based on liposome nanoparticles, they are less stable than polymer particles, have a limited capacity to load antigens, and exhibit poor gastrointestinal stability [50]. The immunostimulation complexes nanoparticle-based vaccine contains hydrophilic antigens that are challenging to incorporate. Antigens are not adequately protected by polymers, and because polymers release antigens prematurely, they offer less antigen protection. Vaccine delivery systems based on inorganic nanoparticles are not biodegradable and have low solubility in water. The vaccine delivery technology based on lipid nanoparticles has low loading efficiency and experiences drug leakage during storage. Exosomes can also be quantified; however, the process is expensive and not sensitive enough [51, 52].

## Challenges in nanomedicine as vaccines

Researchers from around the world have been focusing on the issues associated with nanomedicine and vaccines, particularly the higher production costs of nanodrugs compared to regular drugs. Additionally, hospitals often find it prohibitively expensive to procure these medications, leading to reluctance within the healthcare sector to utilize them. The regulatory aspect of nanomedicine poses the most significant obstacle that needs to be addressed. Changes in the characteristics of vaccines, resulting from nanotechnology, modify their biosafety profile at the nanoscale. Regulation becomes even more challenging when it involves medical devices because distinguishing them from pharmaceutical products at the nanoscale is a more complex task [53, 54].

## Universal-influenza vaccines

The most effective method of preventing influenza infection is vaccination. However, there are other challenging issues when it comes to managing influenza outbreaks, often resulting from changes in antigenic drift and antigenic shift in the virus's genome. These unexpected genomic changes in influenza viruses allow them to evade antibody neutralization [55]. Developing a universal influenza vaccine that offers complete and long-lasting protection is highly desirable to address the limitations and challenges associated with current vaccines. The ideal influenza vaccine would provide protection against all influenza virus subtypes (A and B types), various antigenic variations, and subtypes of HA and NA. To achieve this, the vaccination must induce cross-protective antibodies. This can be accomplished by focusing on conserved epitopes in proteins such as HA, NA, and M2, as well as internal proteins like M1 and NP.

A universal influenza vaccine is considered a promising candidate due to the region of HA known as the stem or stalk region, which plays a significant role in preventing infection among various influenza virus strains [56]. Furthermore, some antibodies isolated from humans that target the viral capture region are capable of neutralizing all influenza A virus subtypes, suggesting their potential use in developing a universal influenza vaccine [57]. For instance, an immune response can be redirected from the head region domain to the capture domain using a progressive chimeric HA vaccination technique (Table 2).

**Table 2:** Universal influenza vaccine candidates in preclinical and clinical trials

Vaccine candidate	Vaccine type	Manufacturer	Clinical phase
**Chimeric HA proteins**	Hemagglutinin based	Glaxo-SmithKline	Phase 1
**(COBRA) Computationally optimized broadly reactive antigens**	Computationally optimized antigens	Sanofi-Pasteur	Preclinical
**NP, M1 and HA peptides (Multimeric-001)**	Recombinant proteins	BiondVax Pharmaceuticals Ltd.	Phase 3

Chimeric HAs are created by combining the stalk domains of group 1 or 2 influenza viruses with the head domains of avian influenza virus subtypes. This universal influenza vaccine candidate was found to be safe and capable of eliciting a broad, potent, persistent, and effective immune response directed towards the conserved, but often overlooked, stalk of the hemagglutinin [58], following the completion of a phase I clinical trial in 2020. Additionally, a broadly reactive antigen (COBRA)-based universal influenza vaccine targeting H1 was developed (Table 2). To assess the breadth of B cell responses, researchers compared antibody-secreting cells induced by previous H1N1 vaccine strains with those induced by a COBRA hemagglutinin, also referred to as P1. Monoclonal antibodies (mAbs) generated in response to P1 HA displayed a wide range of HA recognition, ranging from narrowly reactive to broadly reactive mAbs. Multimeric-001, a novel vaccine containing conserved linear epitopes from the HA, NP, and M1 proteins of influenza A and B strains, is also designed to protect against both seasonal and pandemic influenza virus types (Table 2). In 2020, this vaccine underwent a phase III clinical trial to evaluate its safety, acceptability, and humoral and cellular immune responses [59]. The vaccine was well tolerated, with no significant adverse effects observed.

The cellular and humoral responses demonstrate that the vaccination provides cross-immunity against influenza virus strains without mutations. Compared to individuals who received only the seasonal vaccine, those who received a dose of Multimeric-001 before the seasonal vaccine showed a stronger antibody response against H1N1 and H3N2 strains. Additionally, individuals who received the Multimeric-001 vaccination had higher CD4+ and CD8+ T cell responses to H1N1, H3N2, and influenza B compared to baseline.

### References: [60, 61].

#### Economic benefit for current and future vaccine

In assessing the value of new mRNA and combination influenza/COVID-19 vaccines in low- and middle-income countries, it is essential to examine their cost-effectiveness. Current modeling studies, such as Waterlow et al.’s, have limitations: they focus on medically attended influenza cases but do not consider the full spectrum of influenza's impact. This includes non-respiratory and non-medically attended cases, which are particularly relevant for infants and older adults. Additionally, these models do not account for the broader benefits of influenza vaccines, such as reducing severe disease, preventing non-respiratory complications like heart attacks, or decreasing antibiotic use. To gain a more comprehensive understanding of the advantages of influenza vaccines, we should improve the data used in future modeling assessments [62, 63].

#### Clinical trials

The Vaccine Research Center, part of the National Institute of Allergy and Infectious Diseases (NIAID), has developed a flu vaccine based on nanoparticles. Manufactured by the Frederick National Laboratory for a Phase 1 clinical trial, this vaccine has proven to be safe, well-tolerated, and effective against various flu subtypes. It has the potential to offer longer-lasting and broader protection compared to traditional annual flu shots, possibly leading to a universal flu vaccine [64]. Unlike conventional protein-based flu vaccines produced in chicken eggs, which are time-consuming to make and require yearly updates due to viral mutations, this nanoparticle vaccine is faster to produce and can be quickly adapted to new influenza strains, making it valuable for pandemic response. The research team used a ferritin protein that self-assembles into a scaffold, allowing them to attach viral proteins that trigger an immune response specifically against the H2 subtype of influenza.

The Phase 1 clinical trial involved 50 healthy volunteers aged 18 to 70. The study included individuals with and without prior exposure to the H2 flu subtype to assess the vaccine's effectiveness in both cases. Results indicated that the vaccine was safe and well-tolerated in both groups, and participants developed antibodies, including those targeting a stable region of the virus. This suggests that the nanoparticle vaccine may offer broader and more durable protection compared to standard seasonal flu vaccines.

In conclusion, the Phase 1 trial demonstrated the potential of a new generation of vaccines using orderly arrays of antigens on self-assembling nanoparticles. The results suggest that this ferritin nanoparticle-based vaccine platform could be beneficial for pandemic preparedness and the development of a universal influenza vaccine [65].

#### Vaccine design

##### Production

Vaccine production comprises several distinct stages. The vaccine manufacturing process can be broken down into the following key steps:

1. Antigen generation: initially, the antigen is produced from the virus or microbe. This involves growing the microorganism in various mediums, such as chicken eggs for influenza, cell lines, cultured human cells for hepatitis A, or bioreactors for bacteria like Haemophilus influenzae type b. Proteins or components of the organism can also be generated in yeast, bacteria, or cell cultures. In some cases, bacteria or viruses may be weakened using chemicals or heat to create the vaccine.

2. Antigen isolation: following the generation of the antigen, it is separated from the cells or medium used to produce it. For weakened or attenuated viruses, this may not require further purification. Recombinant proteins, however, often undergo multiple purification steps, such as ultrafiltration and column chromatography, before they are suitable for use.

3. Formulation: once the antigen is obtained, it is formulated into the final vaccine product by adding adjuvants, stabilizers, and preservatives. Adjuvants boost the immune response to the antigen, stabilizers extend the product’s shelf life, and preservatives enable the use of multi-dose vials. Developing combination vaccines can be challenging due to potential incompatibilities and interactions among the antigens and other vaccine components.

To ensure vaccine production meets quality standards, the product must be shielded from exposure to air, water, and potential contamination by humans. Additionally, the production environment should be safeguarded against any spills of antigens [66].

##### Application

Vaccine design has a broad range of practical uses in preventing and managing infectious diseases. Notable applications include:

1. Disease prevention: vaccines are primarily created to prevent infectious diseases by training the immune system to recognize and defend against harmful pathogens. They have played a pivotal role in controlling and even eradicating diseases like polio, smallpox, and measles.
2. Pandemic preparedness: vaccine design is critical in preparing for potential pandemics. Scientists can work on developing vaccines for new and emerging infectious diseases, as seen with the rapid development of COVID-19 vaccines in response to the SARS-CoV-2 virus.
3. Cancer treatment: some vaccines are designed to stimulate the immune system to target cancer cells. For example, the HPV vaccine helps prevent certain cancers.
4. Allergy management: allergen-specific immunotherapy, such as allergy vaccines or shots, is used to reduce allergy symptoms by desensitizing individuals to allergens like pollen or dust mites.
5. Vector-borne diseases: research continues into designing vaccines against diseases transmitted by vectors, like malaria or dengue fever.
6. Vaccines for emerging diseases: in light of new infectious diseases that may emerge, vaccine design is vital for quickly responding to outbreaks and preventing them from becoming global health threats [67].

##### Limitations of vaccine design

1. Identification of antigens: identifying suitable antigens that can stimulate a protective immune response can be challenging for some pathogens.

2. Antigenic variation: some pathogens exhibit high antigenic variability, making it difficult to design effective vaccines that provide long-lasting immunity.

3. Delivery and stability: developing vaccines that are stable and can be easily administered in resource-limited settings can be a challenge.

4. Ethical and regulatory hurdles: vaccine development and testing involve ethical and regulatory challenges, including informed consent and clinical trial standards.

5. Emerging pathogens: rapid vaccine development in response to new or emerging pathogens can be hindered by limited knowledge and a lack of pre-existing platforms [68].

- Theranostic CAR-T cell therapy is used to target solid tumors and fibroblast activation protein-bearing cells, and the development of influenza vaccines. It hints at the potential for a unique strategy to improve both cancer treatment and vaccine effectiveness [69].

The tumor microenvironment (TME) around cancer cells, particularly the presence of cancer-associated fibroblasts (CAFs) expressing fibroblast activation protein (FAP), plays a critical role in cancer progression. Detecting and targeting FAP is of great interest in oncology, with various imaging modalities like SPECT, PET, CT, fluorescence imaging, and MRI being used for this purpose. Chimeric antigen receptor (CAR)-T cells have been effective in treating certain cancers. However, their success in hematological cancers hasn't translated well to solid tumors, necessitating improvements in the approach. There are also some challenges in delivering CAR-T cells to solid tumors through molecular imaging and cell tracking is essential. This CAR-T cell therapy in treating both solid and non-solid tumors highlights current advances and discusses strategies for overcoming hurdles in treatment [70].

While this information relates to optimizing cancer therapy, there is no direct link to influenza vaccines.

## Conclusion

Influenza presents a significant global public health challenge due to widespread viral antigenic drift and shift. The host's immune system is capable of defending against influenza, necessitating annual vaccination updates. Currently, three types of influenza vaccines are available: inactivated influenza vaccines, live-attenuated influenza vaccines, and recombinant hemagglutinin (HA) vaccines.

The formulation of influenza vaccines is complex and typically includes viral antigens, adjuvants, preservatives, and stabilizers to ensure stability and effectiveness. To meet regulatory standards and ensure safety, these vaccines undergo rigorous quality testing during both the production process and upon completion.

Nevertheless, there is a need for further research to address the limitations of current influenza vaccines, such as their limited efficacy, lengthy production processes, and lack of broad cross-protection. Researchers are working on developing new influenza vaccines to enhance efficacy and potentially provide cross-protection against multiple strains, with the ultimate goal of creating a universal influenza vaccine that eliminates the need for annual updates.

## Data Availability

Not applicable.

## Abbreviations

RNA: Ribonucleic acid
DNA: Deoxyribonucleic acid
HA: Hemagglutinin
NA: Neuraminidase
NP: Nucleoprotein
M1: Matrix-1 protein
M2: Matrix-2 protein
PA: Polymerase acidic protein
PB1: Polymerase basic protein 1
PB2: Polymerase basic protein 2
WHO: World Health Organization
IgA: Immunoglobulin A
IgG: Immunoglobulin G
VLP: Viral-like particles
M2e: Matrix-2 protein
mAbs: Monoclonal antibodies
COBRA: Computationally optimized broadly reactive antigen
